# Diagnostic challenge in a patient with nephropathic juvenile cystinosis: a case report

**DOI:** 10.1186/s12882-017-0721-4

**Published:** 2017-09-26

**Authors:** Satomi Higashi, Natsuki Matsunoshita, Masako Otani, Etsuro Tokuhiro, Kandai Nozu, Shuichi Ito

**Affiliations:** 1 Department of Pediatrics, Yokosuka Kyosai Hospital, 1-16 Beigahama Street, Yokosuka City, Kanagawa 238-0011 Japan; 2 Department of Pediatrics, Kobe University Graduate School of Medicine, Kobe, Hyogo Japan; 3 Department of Pathology, Yokohama City University Medical Center, Yokohama, Kanagawa Japan; 4 Department of Pediatrics, Fujisawa City Hospital, Fujisawa, Kanagawa Japan; 5 Department of Pediatrics, Yokohama City University, Graduate School of Medicine, Yokohama, Kanagawa Japan

**Keywords:** Cystinosis, Juvenile, Fanconi syndrome, *CTNS* gene, Cysteamine

## Abstract

**Background:**

Cystinosis is a rare autosomal recessive lysosomal disorder characterized by the accumulation of cystine in lysosomes. Cystinosis is much rarer in Asian than Caucasian populations. There are only 14 patients have with cystinosis alive in Japan. Most cystinosis is the nephropathic infantile form, as indicated by its apparent and severe clinical manifestations, including renal and ocular symptoms. Patients with the nephropathic juvenile form account for 5% of those with cystinosis. Their diagnosis is frequently delayed and difficult because of slower progression to end-stage renal disease and fewer cystine crystals in the cornea. Molecular analysis and a cysteine-binding protein assay should be performed when patients with proximal tubulopathy of an unknown origin are encountered.

**Case presentation:**

A 12-year-old boy had been suffering from Fanconi syndrome since he was 3 years old. He was only recently diagnosed despite repeated ophthalmological examinations. Corneal cystine crystals were found when he was 12 years old, and he was diagnosed with cystinosis by high free cystine content in granulocytes (6.36 nmol half-cystine/mg protein, normal: <0.15). Analysis of the *CTNS* gene showed two novel heterozygous single nucleotide substitutions of c.329G > C and c.329 + 2 T > C. Both were splicing site variants causing exon 6 skipping proven by transcript analysis, although the functional prediction site showed c.329G > C, p.(Gly110Ala) as a benign missense substitution. The patient’s estimated glomerular filtration rate was 66.8 mL/min/1.73 m^2^. He was immediately treated with cysteamine after diagnosis.

**Conclusions:**

Even if no ophthalmological abnormalities are present, nephropathic juvenile cystinosis should be suspected in children with Fanconi syndrome. Transcript analysis was useful to detect pathogenic splicing variants in this patient.

## Background

Cystinosis is an autosomal recessive lysosomal disorder characterized by the accumulation of cystine in lysosomes throughout the body. This results in various clinical manifestations, such as Fanconi syndrome, end-stage renal disease (ESRD), hypothyroidism, hypogonadism, insulin-dependent diabetes mellitus, muscle weakness, swallowing dysfunction, central nervous system complications, keratopathy, and pigmentary retinopathy [1–3]. Cystinosis is caused by mutations in the *CTNS* gene, which encodes the lysosomal cystine carrier protein cystinosin. Cystinosis is classified into three forms according to the age at onset and severity. Nephropathic infantile cystinosis is the most frequent form and patients progress to ESRD in the first decade of life. The nephropathic juvenile form is rare and accounts for 5% of all patients. These patients show much slower progression to ESRD than those with the nephropathic infantile form. Early diagnosis is often difficult and most patients are only diagnosed after they reach 10 years of age [4]. Additionally, non-nephropathic adult cystinosis is associated with photophobia caused by cystine accumulation in the cornea of the eye. The frequency of cystinosis is 1 in 100,000–200,000 in the United States and Europe [1], but is much lower in Asia, including Japan because of the absence of the 57-kb Northern European founder deletion in the *CTNS* gene in Middle Eastern and East Asian people [2, 5]. To date, only seven patients in Japan have received cysteamine therapy for cystinosis. Here we describe a Japanese patient with juvenile nephropathic cystinosis with two novel splice-site mutations causing exon skipping proven by transcript analysis. We also discuss the difficulties in making an early diagnosis of the nephropathic juvenile form.

## Case presentation

The patient was a 12-year-old boy who was the first child of non-consanguineous healthy parents. His growth and development had been normal. He had been followed at another hospital since the age of 3 years because of glycosuria and proteinuria of unknown cause. At the first visit to our hospital, there were no specific physical findings. Laboratory findings showed a low serum uric acid level (2 mg/dL), a high urinary β-2 microglobulin level (78,600 μg/L; normal < 250 μg/L), glycosuria (4+), proteinuria (urinary total protein/creatinine: 1.08 [73 mg/dL/67.1 mg/dL]), and aminoaciduria consistent with Fanconi syndrome. Serum potassium and phosphate levels were within the lower normal limits. Blood gas analysis showed a normal bicarbonate ion value. His estimated glomerular filtration rate (eGFR) was 66.8 mL/min/1.73 m^2^. Levels of serum copper ceruloplasmin, lactic acid, and pyruvic acid indicated that Wilson disease and mitochondrial disease were not the cause of Fanconi syndrome.

**Fig. 1:**
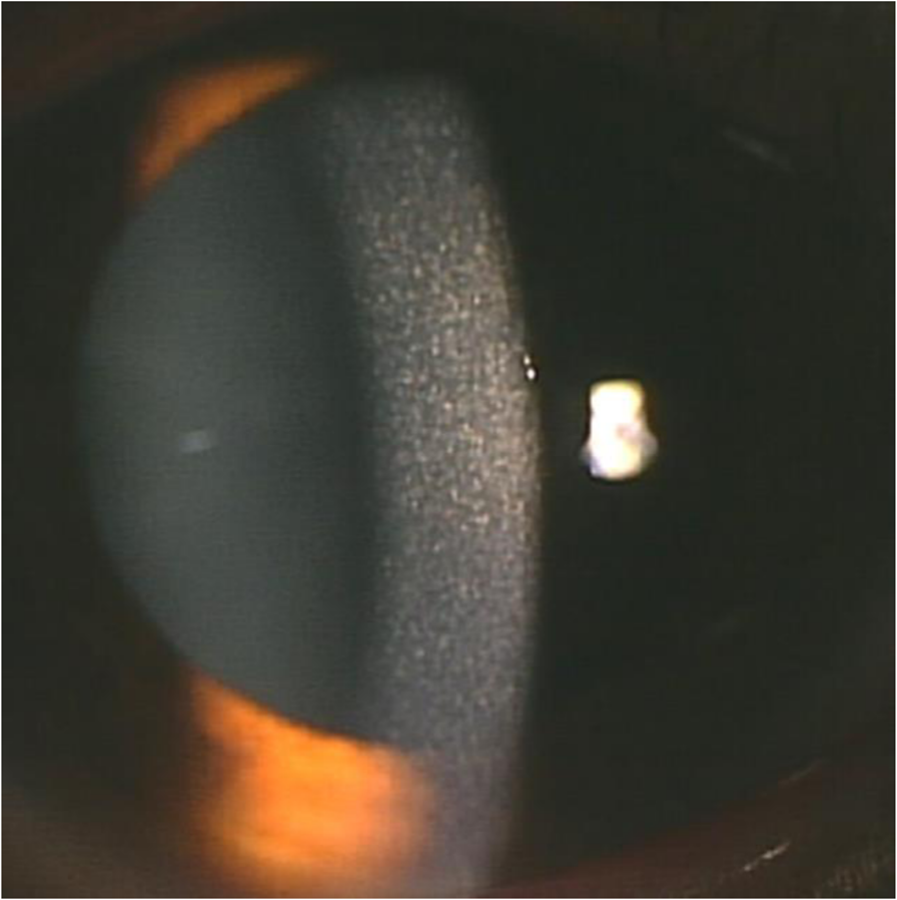
Corneal Slit-lamp examination of the patient. Slit-lamp photograph shows cystine crystals in his cornea

A recent slit-lamp examination showed cystine crystals in the cornea (Fig. 1). He had undergone an ophthalmological examination, including a slit-lamp examination, every 1 or 2 years because of seasonal pollen-allergic conjunctivitis until at least 2 years before referral to our hospital. A cystine-binding protein assay showed a high free cystine content in granulocytes (6.36 nmol half-cystine/mg protein, normal: <0.15). He was finally diagnosed with nephropathic juvenile cystinosis. He had no signs of endocrine involvement, such as hypothyroidism, hypogonadism, or rickets. A renal biopsy showed multinucleated podocytes, tubular atrophy, and interstitial fibrosis. Cystine crystals were detected by electron microscopy. These findings are consistent with cystinosis.

**Fig. 2:**
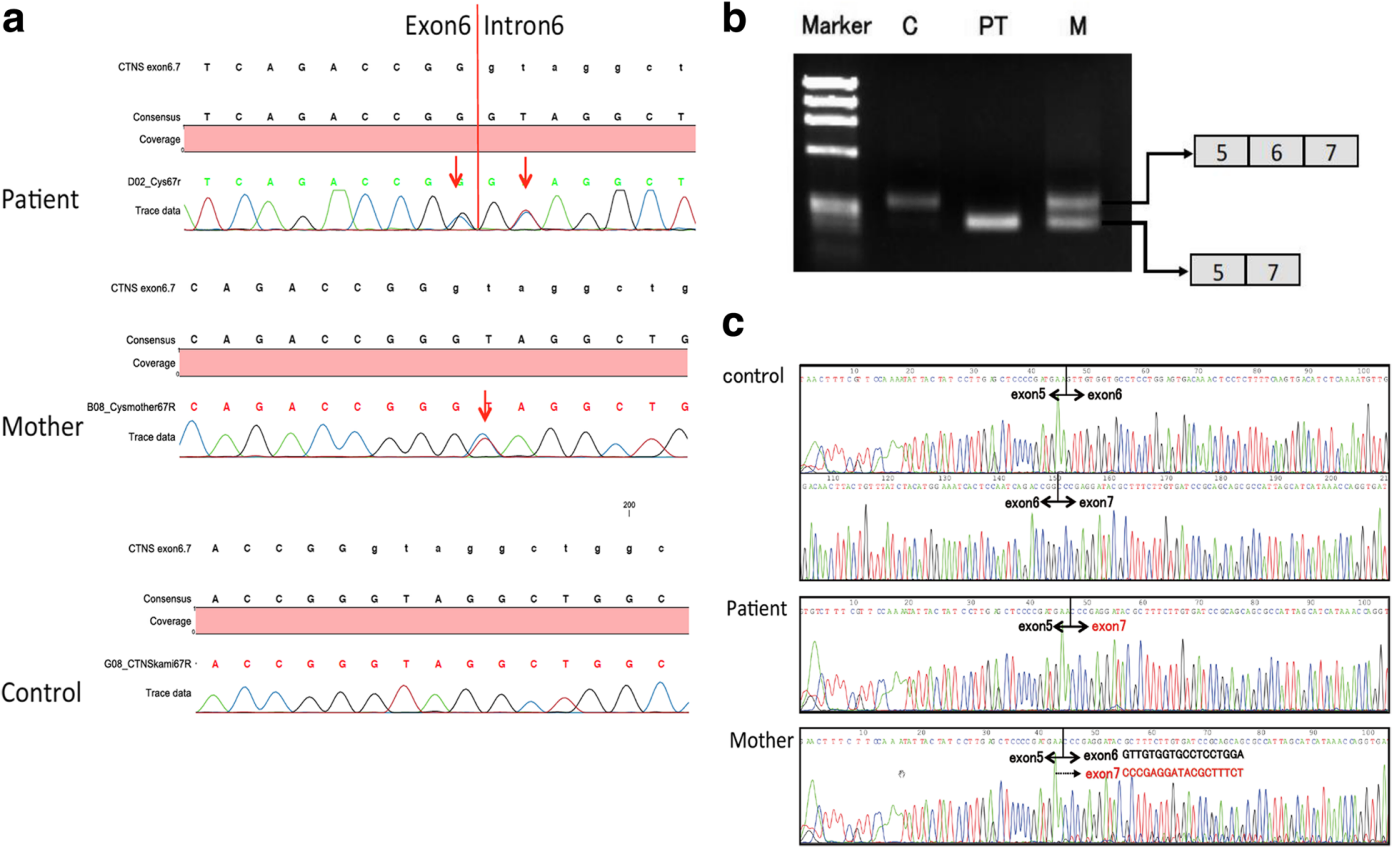
Genetic test results for the CTNS gene. **a** Direct sequencing results for genomic DNA analyses of the patient and mother. The patient possesses two heterozygous mutations of c.329G > C and c.329 + 2 T > C, and the mother only has c.329 + 2 T > C. This finding suggests that c.329G > C is on the paternal allele. **b** Electrophoresis results of transcript PCR products. The patient has a single band, which is smaller compared with the wild-type transcript control. The mother has two bands that are the same and smaller compared with the control. C: control. PT: patient. M: mother. **c** Direct sequencing results of transcripts. The patient showed only a skipped exon 6 sequence and the mother had both sequences with and without exon 6

Direct sequencing analysis of genomic DNA for the *CTNS* gene showed two novel heterozygous single nucleotide substitutions of c.329G > C and c.329 + 2 T > C. The former is the last nucleotide of exon 6 and the latter is at the consensus sequence of the splicing donor site at intron 6 (Fig. 2a). The nucleotide substitution of c.329 + 2 T > C was on the maternal allele. Although the father’s genomic DNA analysis was not available because of the absence of consent, these results could suggest the patient had a compound heterozygous mutation in the *CTNS* gene. The former variant (c.329G > C) might be a single nucleotide polymorphism of p.(Gly110Ala) because the two functional prediction sites of the amino acid variant (PolyPhen2 and SIFT) showed this as benign. However, c.329 is the last nucleotide of exon6 and an effect of the splicing process from the exonic last nucleotide variant is possible. We subsequently conducted transcript analysis using mRNA extracted from leukocytes. cDNA reverse transcribed from mRNA was amplified by nested PCR using the following *CTNS* primer pairs: first PCR forward TTTCCCCTGAAGCTCGTAGA-3′ and reverse 5′-AAGACCCCGAGTCCAAACTT, and second PCR forward TCAGCCTCACTGTTCCTCCT and reverse CGAAGCTCAGACCAATGACA. PCR-amplified products were purified and subjected to direct sequencing. We found that both heterozygous mutations resulted in exon 6 skipping (Fig. 2b, c). The patient’s transcript showed the absence of exon 6 without the missense variant p.Gly110Ala, which indicates that not only the variant c.329 + 2 T > C, but also the substitution c.329G > C, were splicing site variants. This provides strong evidence that the variant c.329G > C is the pathogenic splicing site variant.

The patient was administered cysteamine 1.3 g/m^2^/day immediately after diagnosis to reduce accumulated cystine from lysosomes and prevent further accumulation. Drug administration began with a quarter dose of the target dose and was increased by a quarter dose biweekly. We also administered oral phosphate and potassium citrate/sodium citrate hydrate for treatment of hypophosphatemia and acidosis. Seven months after initiating cysteamine, a cystine-binding protein assay showed significantly decreased free cystine content (1.01 nmol half-cystine/mg protein; therapeutic target: <1.0) in granulocytes at 6 h after taking cysteamine. eGFR improved to 77.8 mL/min/1.73 m^2^ from 66.8 mL/min/1.73 m^2^.

## Discussion

Among the three forms of cystinosis, diagnosis of the nephropathic juvenile form is especially difficult because of the low frequency and mild clinical manifestations compared with the nephropathic infantile form. Delayed diagnosis often results in irreversible renal damage.

Patients with nephropathic infantile cystinosis are born without any complications. At the age of 6–12 months, Fanconi syndrome causes polyuria, thirst, failure to thrive, growth retardation, vomiting, dehydration, constipation, developmental delays, and rickets. Untreated infantile patients rapidly progress to ESRD in the first decade of life [1–5]. Renal transplantation is the best treatment for ESRD because this disease does not recur in the graft organ [6]. However, even after renal transplantation, accumulation of cystine in lysosomes, except for the kidney, continues. Accumulation of cystine causes photophobia and visual impairment starting from the second decade of life. The most frequent endocrine organ involvement is hypothyroidism and hypogonadism found in untreated cystinosis patients older than 10 years. During the third decade of life, encephalopathy may progress and imaging findings show cerebral cortical atrophy [1–3].

There have been few reports of nephropathic juvenile cystinosis. Servais et al. reported 14 patients with the late-onset nephropathic form [7]. The first clinical symptoms were photophobia because of corneal cystine deposits in 10 patients. Corneal deposits in four patients with proteinuria were identified later in life after diagnosis.

A slit-lamp examination is useful for diagnosing nephropathic infantile cystinosis once a patient is 1–2 years old [8], but there are some limitations regarding the nephropathic juvenile form. In our patient, despite repeated slit-lamp examinations, cystine crystals had not appeared in his cornea until he was 10 years old. Cystine-binding protein assays should be performed in children with proximal tubulopathy and Fanconi syndrome of unknown origin, even if ocular manifestations appear absent. Additionally, patients with the nephropathic juvenile form show slow progression of renal involvement and mild urinary abnormalities, such as proteinuria or glycosuria.

A renal biopsy may help diagnosis the nephropathic juvenile form. Our patient showed multinucleated podocytes, cystine crystals, atrophy of tubules, interstitial fibrosis, and glomerular solidification. Among them, multinucleated podocytes suggested the possibility of nephropathic cystinosis, even in the absence of a history of Fanconi syndrome or chronic tubulointerstitial nephropathy [9].

Molecular analysis of the *CTNS* gene is a powerful tool for early diagnosis and can be used for prenatal diagnosis of cystinosis. In our patient, we observed two novel heterozygous mutations. One of these was an apparent pathogenic mutation at a splicing consensus site (c.329 + 2 T > C). The other mutation (c.329G > C, p.(Gly110Ala)) was suggested as a benign variant by two of the most popular prediction sites, PolyPhen2 and SIFT, although it was considered a missense mutation. It is unclear whether c.329G > C single base substitution may lead to a splicing abnormality. This was finally proven as pathogenic by transcript analysis. Therefore, transcript analysis was a good tool in the diagnosis of cystinosis in our patient. Servais et al. reported that four of 14 patients with late-onset nephropathic cystinosis showed only one mutation [7]. Transcript analysis may help find hidden mutations in some patients. Tarante et al. reported that some patients with cystinosis require transcript analysis to find DNA sequencing-undetectable mutations [10].

Among Asians, cystinosis is extremely rare compared with people from the United States and Northern Europe, because the 57-kb deletion is rare in Asians [5]. The two novel mutations in our patient also give some insight into the genetic epidemiology of cystinosis.

Cysteamine is a specific treatment for cystinosis. Cysteamine depletes lysosomal cystine content by reacting with cystine, and forming the mixed disulfide cysteine-cysteamine. Cysteamine treatment does not cure Fanconi syndrome, but prevents or delays progression to ESRD [11]. This treatment prevents the development of multiple extra-renal complications [12]. Early diagnosis and treatment of cystinosis are essential for preventing renal and extrarenal organ damage. Although cysteamine eyedrops are known to be effective at removing or preventing the accumulation of cystine crystals in the cornea, no product has yet obtained pharmaceutical approval in Japan. We are considering making cysteamine eyedrops at our institution for this patient.

Physicians need to recognize the difficulty of early diagnosis and limitations of a slit-lamp examination to identify the nephropathic juvenile form. Patients with the nephropathic juvenile form show slow progression of renal involvement and mild urinary abnormalities, such as proteinuria or glycosuria. Molecular analysis and a cystine-binding protein assay should be performed when patients with proximal tubulopathy of an unknown origin are encountered.

## Conclusion

Proteinuria and glycosuria could indicate nephropathic juvenile cystinosis. A slit-lamp examination may help in the diagnosis of this condition, but cystine crystals may not be observed before adolescence in some cases. To the best of our knowledge, this is the first report of two novel mutations involved in cystinosis, one of which was proven by transcript analysis as pathogenic.

## Abbreviations

eGFR: estimated glomerular filtration
ESRD: End-stage renal disease
